# Comparing strategies for combined testing of rare and common variants in whole sequence and genome-wide genotype data

**DOI:** 10.1186/s12919-016-0042-9

**Published:** 2016-10-18

**Authors:** Dörthe Malzahn, Stefanie Friedrichs, Heike Bickeböller

**Affiliations:** Department of Genetic Epidemiology, University Medical Center, Georg-August University Göttingen, Humboldtallee 32, 37073 Göttingen, Germany

## Abstract

We used our extension of the kernel score test to family data to analyze real and simulated baseline systolic blood pressure in extended pedigrees. We compared the power for different kernels and for different weightings of genetic markers. Moreover, we compared the power of rare and common markers with 3 strategies for joint testing and on marker panels with different densities. Marker weights had much greater influence on power than the kernel chosen. Inverse minor allele frequency weights often increased power on common markers but could decrease power on rare markers. Furthermore, defining the gene region based on linkage disequilibrium blocks often yielded robust power of joint tests of rare and common markers.

## Background

The kernel score test is a global covariate-adjusted multilocus procedure that tests for overall association of sets of markers (see Schaid [1] for a review). This reduces the multiple-testing burden. Tested marker sets can, for example, belong to a pathway or candidate gene. The kernel score test can be applied to common and rare variants alike, as well as to data of genome-wide association studies (GWAS) or sequence data where it is named SKAT (sequence kernel association test). The kernel score test was developed for independent subjects [1]. Recent contributions by others and ourselves [2–6] extended the kernel score test to family data.

The kernel is chosen to describe genetic correlation among subjects. Different kernels have been suggested for genetic epidemiological applications. These kernels differ in whether marker–marker interactions are modeled and how complex the interaction effects may be. A frequent choice is to apply the kernel function on weighted minor allele dosage data (thus using an additive coding of minor allele effects). The dosage weights increase with decreasing minor allele frequency corresponding to the *a priori* assumption that less-frequent variants may have larger effects. Weighting allows rarer variants to contribute more to the overall test despite of their low frequencies.

With appropriate weighting, rare and common variants may be entered together into the kernel for joint testing. Recently however, Ionita-Laza et al. [7] proposed alternatives that can be more powerful. We explored these alternative joint tests on rare and common variants in the Genetic Analysis Workshop 19 (GAW19) family data. Moreover, we compared the power of different marker weights and kernels on sequence and GWAS panels. As we focused on genes, we also explored how size or positioning of a flanking region affects the test power.

## Methods

### Data

We analyzed baseline systolic blood pressure (SBP) and dosage data in the extended Mexican American pedigrees of the GAW19 family data, which are identical to the Genetic Analysis Workshop 18 data [8]. As before [6], we considered subjects with known baseline SBP and baseline diastolic blood pressure, sex, and age, who were not on blood pressure medication (real SBP: 706 subjects, excluding the first listed monozygotic twin of 2 observed twin pairs; simulated SBP: 740 to 781 subjects, numbers vary for 200 simulated study replicates because of inclusion criteria). For real SBP, we considered candidate gene *AGTR1* [9] on chromosome (chr) 3 that tends to associate with SBP in the present family sample [6]. For simulated SBP, we selected from the simulation answers 5 strongly associated genes with various linkage disequilibrium (LD) structures: *MAP4* (very homogeneous LD, chr3) and, in the order of increasing variability of LD, *TNN* (chr1), *FLT3* (chr13), *LEPR* (chr1), and *GSN* (chr9). We used NCBI build 37, International Haplotype Map Project (HapMap) [10] reference data for Mexican Americans and the default algorithm in Haploview 4.2 [11] with a required fraction of strong LD of 0.7 and confidence interval limits of 0.5 and 0.8 to determine LD-blocks based on the D’ measure. Gene regions were defined as the LD-block(s) that contained the gene. For *AGTR1*, we also considered the region from the first to the last exonic position and flanking regions of 30 kb or 500 kb. For the same subjects, we used 2 single-nucleotide polymorphism (SNP) panels: sequence (allele dosage data) and GWAS (allele dosage data reduced to GWAS SNPs). Biallelic SNPs were included for testing if their Hardy-Weinberg equilibrium test *p* values were equal to or greater than 10^−5^ (rounding imputed dosages for this purpose only) and if at least 7 observations of the minor allele were present in the sample. The latter parallels minimum data requirements in parametric regression.

### Kernel score test for family data

Here we briefly summarize our method introduced in [6], denoting vectors and matrices by bold letters. Baseline SBP is right-skewed distributed and was therefore rank-normalized by Blom transformation [12] to standard normally distributed target variables **Y** = (Y_1_,…,Y_n_). **Y** depend on fixed covariate effects **b** (intercept, age, sex, age × sex interaction), random effects **c** that adjust for familial polygenic background, a semiparametric model **h**(**G**) of genetic markers **G**, and regression residuals **e** ~ *N*(0,s^2^ **I**) with residual variance s^2^.

1 $$ \mathbf{Y}=\mathbf{X}{\mathbf{b}}^{\mathrm{T}}+\mathbf{Z}{\mathbf{c}}^{\mathrm{T}}+\mathbf{h}\left(\mathbf{G}\right)+\mathbf{e} $$

**X**, **Z** are the design matrices for fixed covariate effects and random family effects. **h**(**G**) = **Ka** ^T^ depends on a *n* × *n* dimensional kernel matrix **K** of genetic similarities between *n* subjects on markers **G**, and multivariate normally distributed random effects **a** ~ *N*(0,τ**K**) [1]. One tests for a genetic covariance component τ.

The kernel score test is computed from restricted maximum likelihood parameter estimates of the genetic null model (where **h**(**G**) = **0**). Thus, the null model estimates fixed covariate effects **b**_**o**_, random pedigree effects **c**_**o**_, the variance s^2^_fam_ of the polygenic familial component, and the residual variance s^2^_o_. The null model was adjusted for polygenic familial background based on the kinship coefficient matrix Φ_kin_ = **ZZ** ^T^ using R-packages kinship2 and coxme with R-function lmekin. The kernel score test statistic is.

2 $$ \mathrm{Q} = {\mathbf{R}}^{\mathrm{T}}\mathbf{M}\mathbf{R} $$

**R** = **P**_o_^1/2^ **Y** are standard normally distributed residuals and matrix **M =** (**P**_o_^1/2^ **K P**_o_^1/2^)/2 incorporates the kernel [6]. **P**_**o**_ **= V**_**o**_^**−1**^–**V**_**o**_^**−1**^ **X(X** ^**T**^ **V**_**o**_^**−1**^ **X)** ^**−1**^ **X** ^**T**^ **V**_**o**_^**−1**^ is the null projection matrix with **V**_o_ = s^2^_o_ **I** + s^2^_fam_ **ZZ** ^T^. The *p* values for test statistic (2) were calculated by Davies’ exact method [13] with the R package CompQuadForm from sample estimates Q and all eigenvalues of matrix **M**.

### Kernels and single-nucleotide polymorphism weights

We applied all kernel functions on allele dosage data **g**_i,_ **g**_j_ (for pairs of subjects i, j) on N_SNP_ biallelic SNP markers. The kernel matrix entries are

3 $$ \mathrm{Linear}\ \mathrm{kernel}\kern0.5em {\mathbf{K}}_{\mathrm{i}\mathrm{j}}={{\mathbf{g}}_{\mathrm{i}}}^{\mathrm{T}}\mathbf{W}{\mathbf{g}}_{\mathrm{j}} $$

4 $$ \mathrm{Radial}\ \mathrm{basis}\ \mathrm{function}\ \left(\mathrm{R}\mathrm{B}\mathrm{F}\right)\ \mathrm{kernel}\kern0.5em {\mathbf{K}}_{\mathrm{i}\mathrm{j}} = \exp \left(-{\upmu^{-}}^1\cdotp {\left({\mathbf{g}}_{\mathrm{i}}-{\mathbf{g}}_{\mathrm{j}}\right)}^{\mathrm{T}}\mathbf{W}\left({\mathbf{g}}_{\mathrm{i}}-{\mathbf{g}}_{\mathrm{j}}\right)\right) $$

with diagonal weight matrix **W**. The linear kernel (3) does not allow for SNP interactions opposed to the RBF kernel (4), which yields polynomial models. Dosage weights are normed **W**_mm_ = f(ν_m_)/∑_m_f(ν_m_) for any chosen SNP set m = 1,…,N_SNP_ and depend on the minor allele frequency (MAF) ν of the respective SNP. We considered: f(ν_m_) = 1 (treating SNPs alike), f(ν_m_) = 1/ν_m_, as well as f(ν_m_) = *Beta*(ν_m_,1,25) for ν_m_ equal to or less than 5 % and f(ν_m_) = *Beta*(ν_m_,0.5,0.5) for ν_m_ greater than 5 % as suggested earlier [7]. *Beta*-density weights distinguish MAFs more moderately than 1/ν-weights. For the RBF kernel (4), the scale parameter μ was the average weighted squared genetic difference between subjects Σ_i,j_((**g**_i_-**g**_j_)^T^ **W**(**g**_i_-**g**_j_))/n^2^ multiplied by the effective number of independent SNPs in the tested set [14].

### Strategies for combined testing of common and rare variants

By default, the kernel score test, Eq. (2), is performed with a kernel matrix **K**_**all**_ computed on all dosages with a weighting of common and rare SNPs.

In contrast, Ionita-Laza et al. [7] recently suggested computing the kernel separately for rare SNPs (**K**_**rare**_) and for common SNPs (**K**_**common**_), respectively, in a region of interest. Analogous to Eq. (2), this yields matrices **M**_**rare**_, **M**_**common**_, test statistics Q_rare_, Q_common_, and *p* values *p*_rare_, *p*_common_. The null model, **P**_**o**_ and **R** were always the same. The weighted sum test (WS) on common and rare variants has test statistic [7].

5 $$ {\mathrm{Q}}_{\mathrm{WS}} = \left(1\hbox{--} \upvarphi \right)\cdot {\mathrm{Q}}_{\mathrm{rare}} + \upvarphi \cdot {\mathrm{Q}}_{\mathrm{common}} $$

Weight φ = (tr(**M**_**rare**_ **∙M**_**rare**_)/(tr(**M**_**rare**_ **∙M**_**rare**_) + tr(**M**_**common**_ **∙M**_**common**_)))^1/2^ may be chosen such that (1 − φ)∙Q_rare_ and φ∙Q_common_ have the same variance. *P* values are obtained by Davies’ exact method from sample estimates Q_WS_ and all eigenvalues of matrix ((1 − φ)∙**M**_**rare**_ + φ∙**M**_**common**_). Alternatively, Fishers *p* value pooling can be applied.

6 $$ {\mathrm{Q}}_{\mathrm{FISHER}}=-2 \ln \left({p}_{\mathrm{rare}}\right)-2 \ln \left({p}_{\mathrm{common}}\right) $$

Under H_0_, Q_FISHER_/(1 + 0.25∙*cov*) is chi-square distributed with 16/(4 + *cov*) degrees of freedom [7]. With r = tr(**M**_**rare**_∙**M**_**common**_)/(tr(**M**_**rare**_∙**M**_**rare**_)∙tr(**M**_**common**_∙**M**_**common**_))^1/2^, the covariance between *p*_rare_ and *p*_common_ is *cov* ≈ r∙(3.25 + 0.75∙r) for 0 ≤ r ≤1 and *cov* ≈ r∙(3.27 + 0.71∙r) for −0.5 ≤ r ≤0. Only test statistic (6) yields approximate *p* values; all other *p* values are obtained with Davies’ method and are exact.

## Results and discussion

Our test extension to families holds the nominal significance level and correctly adjusts for a polygenic familial variance component (as demonstrated in [6]). Table 1 lists the *p* values obtained for association testing of *AGTR1* on real SBP, considering common SNPs (MAF >5 %) and rare SNPs (MAF ≤5 %) as well as 3 joint tests (default test **K**_**all**_, WS, Fisher). *Beta*-weights (not shown) performed between equal weights and 1/ν-weights. The 1/ν-weight lowered *p* values particularly on common SNPs. *AGTR1* association is suggested by common as well as rare SNPs. Joint testing of rare and common SNPs was beneficial. In particular, WS and Fisher test *p* values were often smaller (and otherwise close to) the smallest *p* value of the separate rare and common SNP tests. When using ad hoc definitions of the *AGTR1* flanking region, Fisher and WS *p* values remained relatively stable and were also smaller compared to the default test **K**_**all**_. However, on the *AGTR1* containing LD-block all joint tests performed highly similar, *p* values were the smallest and also relatively stable regardless of SNP weights and SNP density.

**Table 1:** Analysis of real data: real SBP and candidate gene *AGTR1*

SNP panel	Weight	Common SNPs		Rare SNPs		Joint tests		
		MAF >5 %		MAF ≤5 %		Default	WS	Fisher
		N_SNP_	*p* value	N_SNP_	*p* value	*p* value	*p* value	*p* value
*AGTR1* with no flanking region, positions 148415571–148460795								
GWAS	equal	11	0.189	7	0.097	0.177	0.102	0.101
	1/ν	11	0.113	7	**0.050**	0.054	**0.044**	**0.043**
SEQ	equal	74	0.203	138	0.060	0.173	0.076	0.076
	1/ν	74	0.160	138	0.098	0.083	0.088	0.090
*AGTR1* with 30 kb flanking region, positions 148385571–148490795								
GWAS	equal	30	0.100	12	0.072	0.092	**0.050**	0.052
	1/ν	30	**0.045**	12	0.069	**0.030**	**0.029**	**0.029**
SEQ	equal	198	0.053	300	0.067	**0.047**	**0.030**	**0.032**
	1/ν	198	**0.039**	300	0.172	**0.045**	**0.044**	**0.050**
*AGTR1* with 500 kb flanking region, positions 147915571–148960795								
GWAS	equal	277	0.206	51	**0.048**	0.196	0.061	0.065
	1/ν	277	0.151	51	0.064	0.102	0.059	0.066
SEQ	equal	2170	0.192	2244	0.069	0.173	0.080	0.085
	1/ν	2170	0.157	2244	0.051	0.062	0.057	0.060
*AGTR1* containing LD-block, positions 148344702–148568958								
GWAS	equal	80	0.058	19	0.076	0.055	**0.035**	**0.036**
	1/ν	80	**0.040**	19	0.114	**0.034**	**0.036**	**0.039**
SEQ	equal	499	**0.029**	592	0.106	**0.027**	**0.027**	**0.030**
	1/ν	499	**0.027**	592	0.112	**0.025**	**0.026**	**0.030**

Association of *AGTR1* with real SBP was tested with a linear kernel on minor allele dosage data for GWAS and sequence (SEQ); *p* ≤0.05 bold. N_SNP_ common and rare SNPs, respectively, were combined into joint tests: kernel **K**_**all**_ (default), weighted sum test (WS), and Fisher’s *p* value pooling for correlated *p* values

**Fig. 1:**
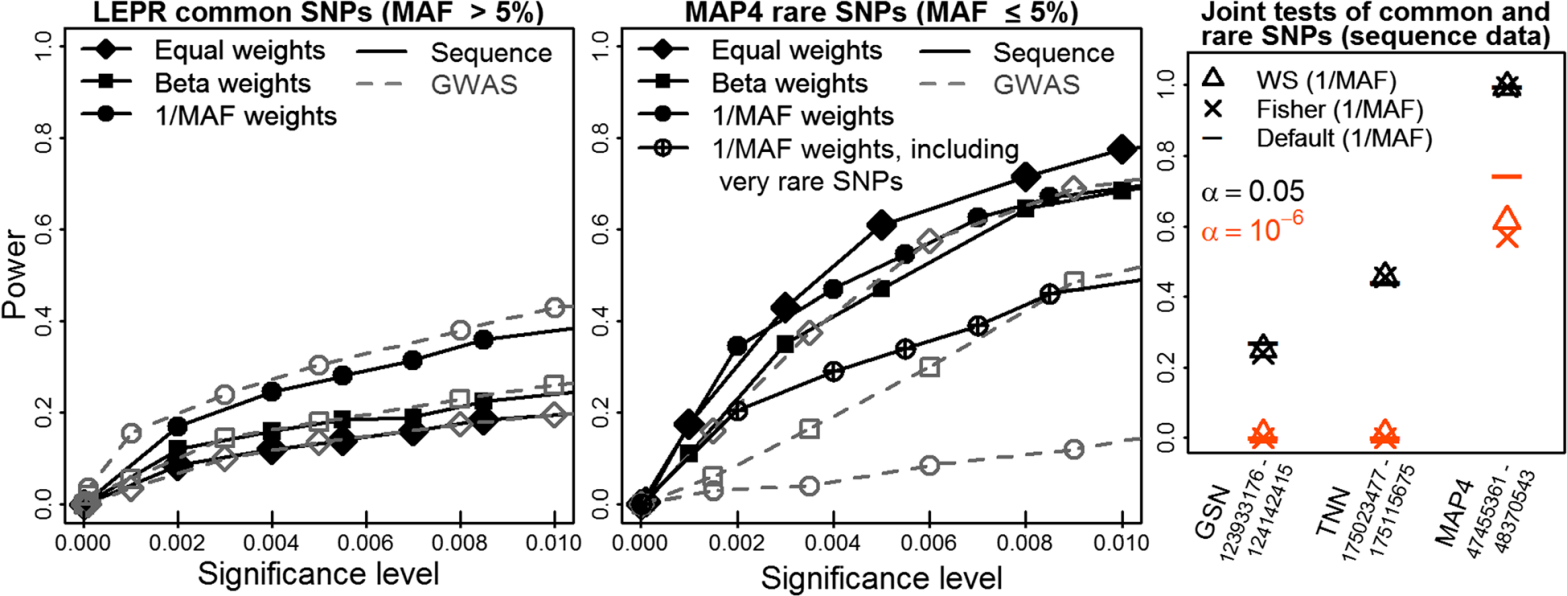
Test power on simulated SBP may greatly depend on SNP weights. *Left* and *middle panels:* Power of the kernel score test over 200 study replicates of simulated SBP as function of the significance level for different SNP weights and SNP panels. *Right panel:* Power of joint tests of rare and common SNPs at 2 significance levels α = 0.05, 10^−6^ when using 1/ν-weights on the sequence of gene-containing LD-blocks. Power estimates for *LEPR* (positions 65743083 to 66106465) and *FLT3* (28490385–28713642) (not shown) were highly similar to *TNN*

Next, we analyzed LD-blocks that contain the genes *MAP4*, *TNN*, *LEPR*, *GSN*, or *FLT3*. Figure 1 displays the average test power on 200 data replicates of simulated SBP. Sequence-derived variants were often more powerful than GWAS with some exceptions (Fig. 1 left and middle panels, black solid lines vs. gray dashed lines). The best were often 1/ν-weights (circle), otherwise equal weights (diamond) were favored. Particularly 1/ν-weights may be beneficial on common SNPs *(LEPR)* and occasionally detrimental on rare SNPs *(MAP4)*. The latter is an exceptional finding but consistent with Table 1 on candidate gene *AGTR1*. On rare *MAP4* SNPs, 1/ν-weights lowered the power, especially when testing also extremely rare SNPs (encircled plus), but less so when testing only MAF equal to or less than 5 % SNPs that had at least 7 observations of the minor allele (filled circle; sequence data). On gene-containing LD-blocks, all joint tests (default test **K**_**all**_, WS, Fisher) often had similar power (Fig. 1, right panel: *LEPR*, *FLT3*, *TNN* with highly similar results [only *TNN* shown]; *GSN* sequence). However, default test **K**_**all**_ was the most powerful test on the gene with homogeneous strong LD (*MAP4*: sequence [Fig. 1, right] and GWAS [not shown]) and on the gene with the most variable LD structure (*GSN*: when using GWAS SNPs, not shown). Then, **K**_**all**_ likely exploited SNP correlations better. When LD-blocks were enlarged by flanking regions, WS and Fisher often were slightly more powerful than **K**_**all**_ (results not shown). The linear kernel had always similar or better power than the RBF kernel (results not shown).

## Conclusions

As the power of kernel methods increases through the exploitation of SNP correlations [2], this ability should be utilized fully by analyzing LD-blocks. SNP weights have a far greater impact on test power than the kernel chosen. Currently, the benefit of 1/ν-weights may be underestimated for common SNPs. On rare SNPs, 1/ν-weights often improve power, but can also be detrimental. Findings are consistent with both real and simulated data. Our results suggest using 1/ν-weights on all SNPs in a single kernel **K**_**all**_ testing LD-blocks and only SNPs with sufficient minor allele observations. Alternatively, one may use WS with 1/ν-weights on common SNPs and equal weights on rare SNPs in the kernels. WS upweights the rare variant contribution globally; see Eq. (5).

## References

[1] Schaid DJ (2010). Genomic similarity and kernel methods I: advancements by building on mathematical and statistical foundations. Hum Hered.

[2] Schifano ED, Epstein MP, Bielak LF, Jhun MA, Kardia SL, Peyser P, Lin X (2012). SNP set association analysis for familial data. Genet Epidemiol.

[3] Chen H, Meigs JB, Dupuis J (2013). Sequence kernel association test for quantitative traits in family samples. Genet Epidemiol.

[4] Oualkacha K, Dastani Z, Li R, Cingolani PE, Spector TD, Hammond CJ, Richards JB, Ciampi A, Greenwood CM (2013). Adjusted sequence kernel association test for rare variants controlling for cryptic and family relatedness. Genet Epidemiol.

[5] Huang J, Chen Y, Swartz MD, Ionita-Laza I (2014). Comparing the power of family-based association test for sequence data with applications in the GAW18 simulated data. BMC Proc.

[6] Malzahn D, Friedrichs S, Rosenberger A, Bickeböller H (2014). Kernel score statistic for dependent data. BMC Proc.

[7] Ionita-Laza I, Lee S, Makarov V, Buxbaum JD, Lin X (2013). Sequence kernel association tests for the combined effect of rare and common variants. Am J Hum Genet.

[8] Almasy L, Dyer TD, Peralta JM, Jun G, Wood AR, Fuchsberger C, Almeida MA, Kent JW, Fowler S, Blackwell TW (2014). Data for Genetic Analysis Workshop 18: human whole genome sequence, blood pressure, and simulated phenotypes in extended pedigrees. BMC Proc.

[9] Baudin B (2005). Polymorphism in angiotensin II receptor genes and hypertension. Exp Physiol.

[10] The International HapMap Consortium (2003). The International HapMap project. Nature.

[11] Barrett JC, Fry B, Maller J, Daly MJ (2005). Haploview: analysis and visualization of LD and haplotype maps. Bioinformatics.

[12] Blom G (1958). Statistical estimates and transformed beta variables.

[13] Davies RB (1980). Algorithm AS 155: the distribution of a linear combination of chi-2 random variables. J R Stat Soc: Ser C: Appl Stat.

[14] Cheverud JM (2001). A simple correction for multiple comparisons in interval mapping genome scans. Heredity (Edinb).

